# Flavonoid Library Screening Reveals Kaempferol as a Potential Antiviral Agent Against African Swine Fever Virus

**DOI:** 10.3389/fmicb.2021.736780

**Published:** 2021-10-21

**Authors:** Erik Arabyan, Astghik Hakobyan, Tamara Hakobyan, Rafaella Grigoryan, Roza Izmailyan, Aida Avetisyan, Zaven Karalyan, Joshua A. Jackman, Fernando Ferreira, Charles C. Elrod, Hovakim Zakaryan

**Affiliations:** ^1^Laboratory of Antiviral Drug Discovery, Institute of Molecular Biology of NAS, Yerevan, Armenia; ^2^Laboratory of Cell Biology and Virology, Institute of Molecular Biology of NAS, Yerevan, Armenia; ^3^School of Chemical Engineering, Sungkyunkwan University, Suwon, South Korea; ^4^Faculdade de Medicina Veterinária, Centro de Investigação Interdisciplinar em Sanidade Animal, Universidade de Lisboa, Avenida da Universidade Técnica, Lisboa, Portugal; ^5^Natural Biologics Inc., Newfield, NY, United States; ^6^Department of Animal Science, Cornell University, Ithaca, NY, United States

**Keywords:** flavonoid, screening, kaempferol, antiviral, autophagy

## Abstract

Naturally occurring plant flavonoids are a promising class of antiviral agents to inhibit African swine fever virus (ASFV), which causes highly fatal disease in pigs and is a major threat to the swine industry. Currently known flavonoids with anti-ASFV activity demonstrate a wide range of antiviral mechanisms, which motivates exploration of new antiviral candidates within this class. The objective of this study was to determine whether other flavonoids may significantly inhibit ASFV infection *in vitro*. We performed a cell-based library screen of 90 flavonoids. Our screening method allowed us to track the development of virus-induced cytopathic effect by MTT in the presence of tested flavonoids. This screening method was shown to be robust for hit identification, with an average Z-factor of 0.683. We identified nine compounds that inhibit ASFV Ba71V strain in Vero cells. Among them, kaempferol was the most potent and exhibited dose-dependent inhibition, which occurred through a virostatic effect. Time-of-addition studies revealed that kaempferol acts on the entry and post-entry stages of the ASFV replication cycle and impairs viral protein and DNA synthesis. It was further identified that kaempferol induces autophagy in ASFV-infected Vero cells, which is related to its antiviral activity and could be partially abrogated by the addition of an autophagy inhibitor. Kaempferol also exhibited dose-dependent inhibition of a highly virulent ASFV Arm/07 isolate in porcine macrophages. Together, these findings support that kaempferol is a promising anti-ASFV agent and has a distinct antiviral mechanism compared to other anti-ASFV flavonoids.

## Introduction

African swine fever (ASF) is a highly contagious hemorrhagic disease that affects wild (*Sus scrofa*) and domestic (*Sus* domesticus) pigs of all breeds and is caused by African swine fever virus (ASFV). Pigs infected with highly virulent ASFV strains develop severe clinical manifestations, such as hemorrhagic lesions, enlargement of multiple organs, including lymph nodes, spleen, kidneys, and liver, anorexia, vomiting, watery to bloody diarrhea, severe depression, huddling, and incoordination (Salguero, 2020). Animals usually die in less than 2weeks with mortality rates reaching up to 100% (Salguero, 2020). Over the last years, more than 8,000 ASF outbreaks have been reported in 25 countries, mostly in Europe and Asia (Wu et al., 2020). While there has been some progress on the ASFV vaccine development front, widespread vaccination remains a long-term objective and ASFV is a serious and immediate threat to the global pig industry (Zakaryan and Revilla, 2016; Sánchez et al., 2019; Bosch-Camós et al., 2020; Wu et al., 2020). These challenges further underscore the need to develop antiviral drugs for the treatment and prevention of ASFV infection (Arabyan et al., 2019).

In terms of structural features, ASFV is a large double-stranded DNA virus of the genus *Asfivirus* within the *Asfarviridae* family. The virion has a complex, double-envelope structure with an overall diameter around 200nm. The viral genome is around 180 kbp and encodes approximately 160 open reading frames depending on the ASFV strain. In pigs, the primary target cells for ASFV replication are monocytes and macrophages, and receptor-mediated endocytosis appears to be the main entry mechanism (Alcamí et al., 1989; Galindo et al., 2015), while a less specific mechanism, micropinocytosis, has also been reported (Sánchez et al., 2012). Upon endocytosis, entering ASFV particles are transported along the endolysosomal pathway where decapsidation occurs (Hernáez et al., 2016). After decapsidation, fusion of the internal virion membrane with the endosomal membrane takes place, followed by the release of ASFV DNA into the cytoplasm where viral factories form and ASFV replication takes place (Simões et al., 2019). Once new ASF virions are assembled, they are transported from viral factories to the plasma membrane through a microtubule-mediated mechanism (Jouvenet et al., 2004). Membrane budding allows newly produced virions to exit host cells, and the extracellular virions acquire an additional lipid membrane through this budding process. One ASFV infection cycle, including attachment, entry, replication, transport, and budding, takes around 24h.

We have previously shown that several flavonoids were able to significantly inhibit ASFV infection *in vitro* by targeting different stages of the viral life cycle (Hakobyan et al., 2016, 2019; Arabyan et al., 2018). Our results prompted us to screen a flavonoid library consisting of 90 compounds in order to identify additional flavonoids with anti-ASFV activity. Here, we performed a cell-based high-throughput screening using the ASFV Ba71V strain and Vero cells. Following validation of top hits, kaempferol was selected for further studies due to its observed antiviral potency against ASFV infection. Then, various experiments were conducted to understand the possible mechanism of action of the anti-ASFV activity of kaempferol.

## Materials and Methods

### Cells, Viruses, and Compounds

Vero (African green monkey kidney) cells were grown at 37°C in Eagle’s minimum essential medium (EMEM; Lonza, Switzerland) supplemented with 10% fetal bovine serum (Lonza, Switzerland), 2mm L-glutamine (Lonza, Switzerland), 100IU/ml penicillin (Reyoung, China), and 100μg/ml streptomycin (Arterium, Ukraine). In experiments with Vero cells, the Vero-adapted ASFV Ba71V strain was used and the viral titration was measured by cytopathic effect (CPE-based) assay on Vero cells. The titer was calculated using Spearman-Kärber endpoint method and expressed as log TCID_50_/ml.

Highly virulent ASFV Arm/07 isolate was used in experiments with porcine alveolar macrophages. Preparation of porcine macrophages was done as previously described (Carrascosa et al., 2011). Porcine macrophages were maintained at 37°C in Dulbecco’s modified Eagle’s medium (Sigma-Aldrich, Germany) supplemented with 10% fetal bovine serum, 2mML-glutamine, 100IU/ml penicillin, and 100μg/ml streptomycin. The titration of ASFV Arm/07 isolate was performed by hemadsorption (HAD) assay and expressed as log HADU_50_/ml.

The library of flavonoids consisting of 90 compounds (Supplementary Table S1) was purchased from ChemFaces (China). Each compound was dissolved in dimethyl sulfoxide (DMSO) as a 5mg/ml stock solution and diluted in EMEM. Dilutions in cell culture medium were performed with the final concentration of DMSO not exceeding 1% (v/v).

### Library Screening

Confluent cells in 96-well cell culture plates (seeding density: 2×10^4^ cell/well) were infected with ASFV Ba71V strain (0.2 TCID_50_/cell) and immediately treated with flavonoids at 20μg/ml concentration, except for 7,8-benzoflavone, diosmin, and khellin that were used at 10μg/ml concentration for 72h at 37°C. After incubation, when the full CPE developed in untreated wells, the cells were evaluated by MTT assay. Briefly, Vero cells were washed with cold PBS and MTT solution [3-(4,5-dimethylthiazol-2-yl)-2,5-diphenyltetrazolium bromide, Sigma-Aldrich, Germany] was added. Then, cells were incubated at 37°C for 2h after adding MTT solution followed by purple formazan extraction by MTT solvent (DMSO). The colorimetric measurements of optical density (OD) were performed on a microplate reader at 570nm (BioTek Epoch2, United States). The percentage of CPE inhibition was calculated by the formula: $\;\left(\frac{{\mathrm{OD}}_{tv}-{\mathrm{OD}}_{v}}{{\mathrm{OD}}_{C}-{\mathrm{OD}}_{v}}\right)*100$ , where OD refers to the optical density of each well, *tv* refers to the flavonoid-treated ASFV-infected cells, *c* refers to mock-infected cells, and *v* refers to the ASFV-infected cells.

ASFV-infected cells treated with DMSO (< 0.5%) served as a negative control, whereas ASFV-infected cells treated with 20μg/ml apigenin served as a positive control. Since each plate contained six negative and six positive controls, their MTT OD values were used to calculate the Z-factor, a statistical parameter that measures the variability of the screening assay (Zhang et al., 1999). The Z-factor was calculated using the following formula: $1-\frac{3\sigma_{p}+3\sigma_{n}}{|\mu_{p}-\mu_{n}|}$ , where σ refers to the standard deviation, μ refers to the mean, *p* refers to the positive control, and *n* refers to the negative control. If the Z-factor value is between 0.5 and 1.0, then the screening assay is considered as an excellent assay (Zhang et al., 1999).

### Cytotoxicity

The cytotoxicity of selected flavonoids was studied in Vero cells and porcine macrophages by the crystal violet staining method (Feoktistova et al., 2016). Confluent cells (seeding density: 2×10^4^ cell/well) were exposed to flavonoids at increasing concentrations ranging from 2.5 to 20μg/ml and incubated for 24h at 37°C in 5% CO_2_. After incubation, the cell culture medium was removed and 4% crystal violet solution (in ethanol) was added to the wells and incubated for 40min at room temperature. Then, the wells were carefully washed with distilled water and 200μl methanol was added to each well for 20min. The OD of each well was measured at 570nm with a plate reader (BioTek Epoch2, United States). The percentage of viable cells was calculated for each concentration by the following formula: $\left(\frac{{\mathrm{OD}}_{t}}{{\mathrm{OD}}_{c}}\right)*100$ , where OD_t_ and OD_c_ correspond to the absorbance of treated and control cells, respectively. The 50% cell cytotoxicity (CC_50_) was calculated by a nonlinear regression analysis of dose–response curves generated from the data.

### Yield Reduction Assay

Vero cells grown in 24-well cell culture plates (seeding density: 2×10^5^ cell/well) were infected with the ASFV Ba71V strain (0.2 TCID_50_/cell) and treated with selected flavonoids at 20μg/ml or 10μg/ml (for 7,8-benzoflavone, diosmin, and khellin) concentrations. The infection was allowed to proceed for 24h. Then, the supernatant was collected and titrated by CPE-based assay.

### Virucidal Assay

The virus suspension containing 2×10^5^ TCID_50_ ASFV particles was incubated with volume solutions of tested compounds of compounds (10μg/ml and 20μg/ml) for 1h at 20°C. Then, Vero cells in 96-well cell culture plates (seeding density: 2×10^4^ cell/well) were infected with the 20-fold dilutions of the treated viral suspension to abolish the possible virostatic effect of the test compounds on ASFV infection. The supernatant was collected and titrated by CPE-based assay after 24h post-infection.

### Dose- and MOI-Dependent Assays

For dose-dependent assay, Vero cells (seeding density: 2×10^5^ cell/well) or macrophages (seeding concentration: 4×10^4^ cell/well) grown in 24-well cell culture plates were infected with ASFV Ba71V strain (0.2 TCID_50_/cell) or ASFV Arm/07 isolate (0.5 HADU_50_/cell), respectively, and treated with selected flavonoids at decreasing concentrations from 20μg/ml to 1.25μg/ml for the ASFV Ba71V strain and from 20μg/ml to 5μg/ml for the ASFV Arm/07 isolate. The infection was allowed to proceed for 24h, and then, the supernatant was collected and titrated by CPE-based assay or HAD assay.

For MOI-dependent assay, Vero cells grown in 24-well cell culture plates (seeding density: 2×10^5^ cell/well) were incubated with the ASFV Ba71V strain with increasing MOIs from 0.1 TCID_50_/cell to 1 TCID_50_/cell and treated with 20μg/ml tested compound. Then, the supernatant was collected and titrated by CPE-based assay at 24h post-infection.

### Time-of-Addition and Time-of-Removal Assays

For time-of-addition assay, Vero cells (seeding density: 2×10^5^ cell/well) or macrophages (seeding density: 4×10^4^ cell/well) grown in 24-well cell culture plates were designated as −2, 0, 2, 4, 8, 12, and 16h, according to the time of ASFV infection. In the pre-infection experiment, Vero cells or macrophages were treated with 20μg/ml flavonoid at 2h before (−2h) infection with ASFV Ba71V (0.2 TCID_50_/cell) or ASFV Arm/07 isolate (0.5 HADU_50_/cell), respectively. In the co-infection experiment, Vero cells were exposed to 20μg/ml flavonoid at the same time that ASFV Ba71V (0.2 TCID_50_/cell) was added to the cells. In post-infection experiment, cells were infected with ASFV Ba71V (0.2 TCID_50_/cell) and the compound was added at 2, 4, 8, 12, or 16h post-infection. The supernatant was collected at 24h post-infection and titrated by CPE-based assay.

For time-of-removal assay, Vero cells grown in 24-well cell culture plates (seeding density: 2×10^5^ cell/well) were infected with the ASFV Ba71V (0.2 TCID_50_/cell) and treated with 20μg/ml flavonoid. Then, the compound was removed at incubation time intervals of 2, 4, 8, 12, or 16h post-infection by washing cells with 1×PBS and replacing the media. The supernatant was collected and titrated at 24h post-infection.

### Virus Entry Assay

In the virus attachment experiments, Vero cells (seeding density: 2×10^5^ cell/well) or macrophages (seeding density: 4×10^4^ cell/well) grown in 24-well cell culture plates were incubated with the ASFV Ba71V strain (0.2 TCID_50_/cell) or ASFV Arm/07 isolate (0.5 HADU_50_/cell), respectively, and the tested flavonoid at 4°C for 1h to allow virus binding but prevent viral internalization. The unbound virus and compound were then replaced by thoroughly washing the cells with 1× PBS, and then, fresh medium containing 3% FBS was added. The virus in supernatant was collected and titrated at 24h post-infection.

In the internalization experiments, Vero cells or macrophages at indicated concentrations were incubated with ASFV Ba71V or ASFV Arm/07, respectively, at 4°C for 1h. Then, the unbound virus was replaced by thoroughly washing the cells with 1×PBS. Test compounds were then added to the cells and incubated at 37°C to allow virus entry to proceed for 1h. Then, the flavonoid was removed by washing with 1×PBS to prevent its action on the later stages of infection. The infection was allowed to proceed for 24h. Then, the supernatant was collected and titrated.

### Liquid Plaque Assay

Vero cells cultured in 6-well plates (seeding density: 4×10^5^ cells/well) were treated with tested compound (10μg/ml and 20μg/ml) and infected with ASFV Ba71V (200 PFU/well) at 37°C. After 1h post-infection, the inoculums were removed and infected/treated cells were cultured in liquid growth medium (EMEM) containing DMSO (as control) or tested flavonoid. In another condition, the compound was added after 1h post-infection and remained in the growth medium throughout the experiment. At 4days post-infection, cells were fixed and stained with crystal violet and then photographed.

### Quantification of ASFV DNA in Viral Factories

Vero cells (seeding density: 3×10^5^ cell/well) or macrophages (seeding density: 2×10^5^ cell/well) grown on coverslips in 12-well cell culture plates were infected with ASFV Ba71V (0.5 TCID_50_/cell) or ASFV Arm/07 isolate (0.5 HADU_50_/cell), respectively, and exposed to the compound (20μg/ml) at 1h post-infection. At 14h post-infection, cells were fixed in a 96% ethanol solution for 30min and stained in fresh Schiff’s reagent (DNA hydrolysis in 5N hydrochloric acid, 60min at 22°C) by the Feulgen method. The cytometric equivalent of DNA content of viral factories was measured by computer-equipped microscope-cytometer SMP 05 (Carl Zeiss, Germany) at 575nm and expressed as an integrated optical density. The measurement was carried out for 100 viral factories per sample.

### Western Blotting Analysis

Vero cells grown in 30mm dishes were infected with ASFV Ba71V at an MOI of 2 or 5, after an adsorption period of 1h. Following this step and before protein extraction, mock- and ASFV-infected cells either alone or exposed to the tested compound (10μg/ml and 20μg/ml) were washed twice with PBS and further processed as published (Freitas et al., 2018). Briefly, protein concentration collected from cell lysate at 16h post-infection was measured with a BCA^™^ protein assay kit (Pierce, Rockford, IL) by following the manufacturer’s guidelines. Ten micrograms of total protein was loaded per well (1mg/ml). Proteins were subjected to SDS-PAGE electrophoresis and electroblotted onto nitrocellulose membranes (Merck KGaA, Darmstadt, Germany) that were incubated with two primary antibodies developed in the laboratory (mouse anti-ASFV-pA104R, 1:100; rabbit anti-β-actin, 1:1000, 13E5, Cell Signaling Technology), at room temperature for 1h, followed by three wash steps with PBST (10min). Then, membranes were incubated with secondary antibodies conjugated with HRP (anti-mouse IgG, 1:30000, 1,010–05; anti-rabbit IgG, 1:10000, 4,010–05; both from Southern Biotech), also for 1h at room temperature. All antibody dilutions were performed in blocking solution (5% w/v BSA) and incubated according to the manufacturer’s recommendations. Finally, protein detection was performed using a chemiluminescence detection kit (Immun-Star HRP Kit, Bio-Rad Laboratories, Hercules, CA, United States), on an Amersham Hyperfilm ECL (GE Healthcare, Piscataway, NJ, United States). The antibody against ASFV-pA104R was previously validated (Freitas et al., 2019).

### Autophagy Detection

Autophagic vacuoles were quantified by using the Autophagy detection kit (ab139484, Abcam, United Kingdom) according to the manufacturer’s protocol. Briefly, Vero cells were grown in 96-well cell culture plates (seeding density: 2×10^4^ cells/well) in cell culture medium without Phenol Red. Cells were infected with ASFV Ba71V (0.2 TCID_50_/cell) and treated with compounds at indicated concentrations in Phenol Red free medium containing 1μl/ml Green Detection Reagent and 1μl/ml Nuclear stain. After 12h or 24h post-infection, green dye signal was analyzed by a fluorescent plate reader (Excitation ~480nm, Emission ~530; Synergy LX, BioTek, United States).

### Statistics

All experiments were conducted in triplicate. Data are expressed as mean±SD of three independent experiments. Data were analyzed by Student’s *t* test. *p*<0.05 was considered to be statistically significant.

## Results

### Screening of Flavonoids Library for Inhibitors Against ASFV

**Figure 1:**
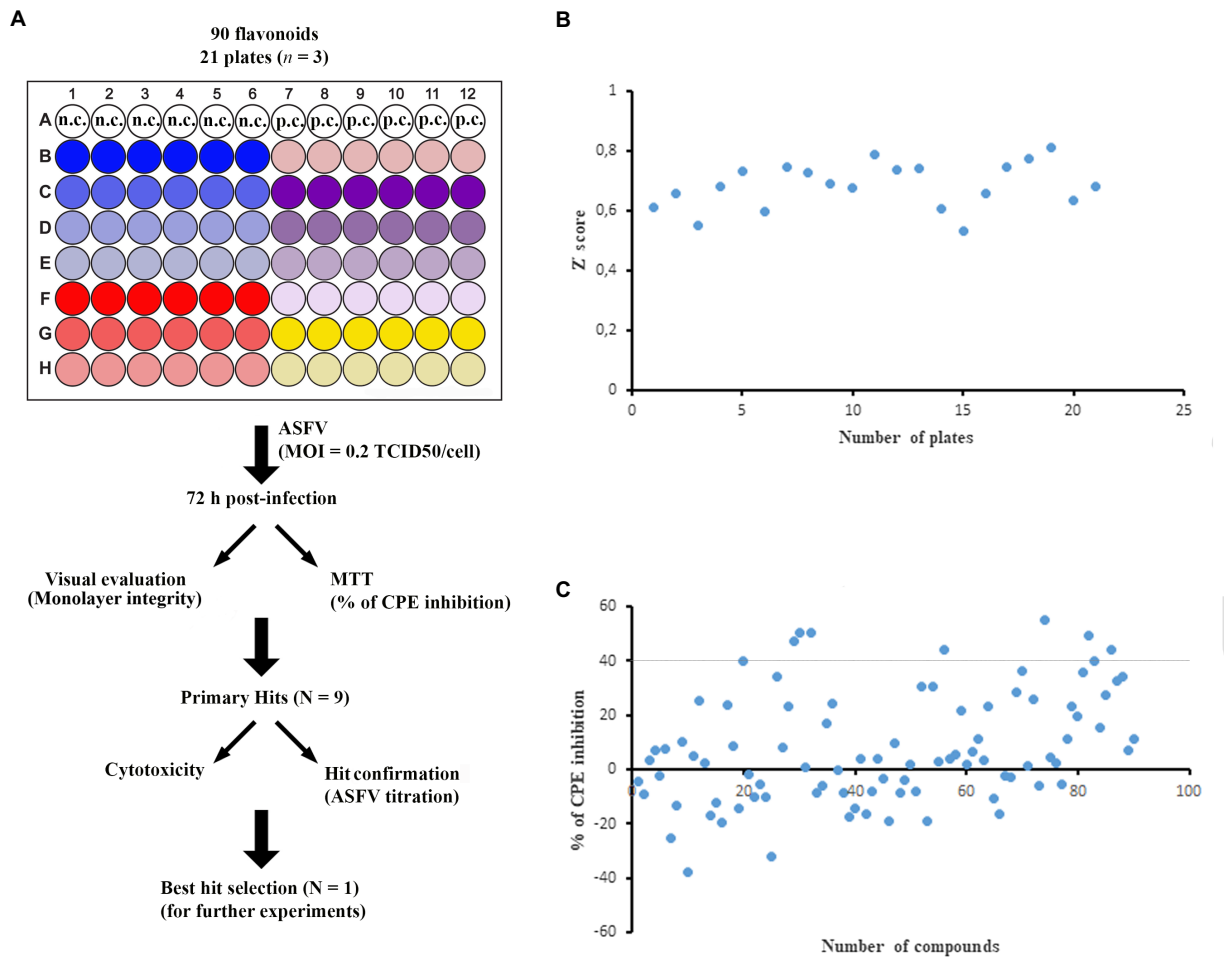
Cell-based library screening. **(A)** Schematic representation of screening outline. ASFV-infected Vero cells were treated with each compound at indicated concentration (6 wells per compound). DMSO was dispensed into wells as a negative control, whereas apigenin was used as a positive control. After 72h post-infection, all wells were visually monitored and stained with MTT. n.c. and p.c. stand for negative control and positive control, respectively. **(B)** Plot of the Z' score of each plate. The Z' score was calculated by using maximum (DMSO; negative control) and minimum (apigenin; positive control) MTT values. **(C)** Inhibition of viral CPE by tested compounds. Compounds that inhibited ASFV-induced CPE by ≥40% were selected for further validation. All experiments were conducted in triplicate. The standard deviation of each compound was not included in the figure for presentation clarity.

We screened a 90-flavonoid library (Supplementary Table S1) from ChemFaces (China) by using a cell-based colorimetric assay that allowed us to track the development of CPE by MTT (Figure 1A). We used a single compound concentration (20μg/ml) in the library screening experiments, except for 7,8-benzoflavone, diosmin, and khellin (10μg/ml) due to their cytotoxicity at the higher concentration. Each screening plate contained negative control wells (cell culture medium + DMSO <0.5%) and positive control wells (apigenin as a validated inhibitor of ASFV; Hakobyan et al., 2016). We used the maximum (positive control) and minimum (negative control) MTT OD values from each plate to generate Z' values. As shown in Figure 1B, the Z' value of each plate was higher than 0.5, yielding an average of 0.683 for the entire screening process. This value indicates that the assay has high consistency and is robust for the screening of ASFV inhibitors (Zhang et al., 1999). Then, we selected primary hits based on the following criteria: (1)≥40% inhibition of ASFV-induced CPE and (2) no cell monolayer damage upon treatment with the flavonoid. Nine flavonoids, 7,8-benzoflavone, calycosin, diosmin, isosinensetin, kaempferol, khellin, maackiain, sakuranetin, and sinensetin displayed more than 40% inhibition of ASFV-induced CPE with no apparent cell monolayer damage (Figure 1C). Therefore, those nine flavonoids were selected for further downstream validation.

### Validation of Selected Compounds

**Figure 2:**
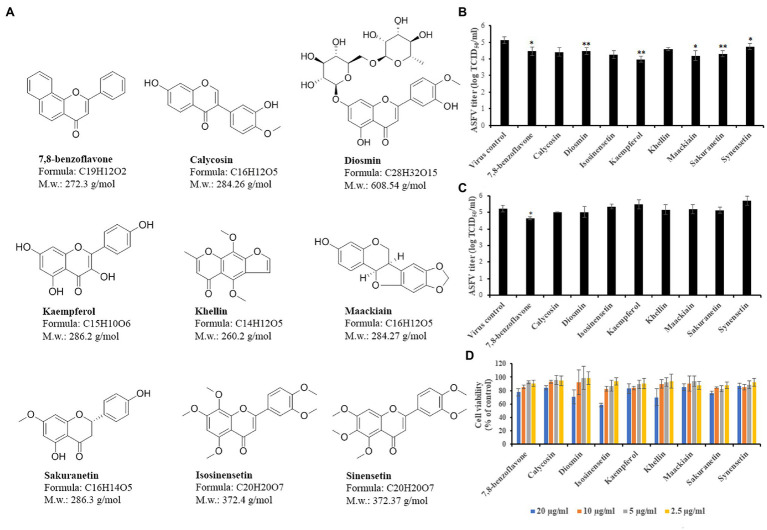
Validation of selected flavonoids. **(A)** Nine top compounds selected for validation. **(B)** ASFV titer in Vero cells upon treatment with selected compounds at indicated concentrations. **(C)** Direct (virucidal) effect of compounds on extracellular ASFV particles. **(D)** Cytotoxicity of compounds on Vero cells evaluated by crystal violet staining method. Results represent the mean (± SD) of three independent experiments (*n*=3). Significant differences compared to control are denoted by ^*^p<0.05 and ^**^p<0.01.

To validate the antiviral activity of selected flavonoids, we performed virostatic, virucidal, and cytotoxicity experiments (Figure 2A). For virostatic experiments, we infected Vero cells with ASFV and treated them with a single dose of compounds, simultaneously. After 24h post-infection, when the first cycle of ASFV infection was completed, we harvested the virus for titration. As shown in Figure 2B, out of the nine selected compounds, six flavonoids demonstrated statistically significant antiviral effects on ASFV infection. The most potent inhibitor, kaempferol, reduced the viral yield from 5.14±0.19 log TCID_50_/ml to 3.98±0.19 log TCID_50_/ml (*p*<0.01), thereby inhibiting ASFV infection by more than 92%. For virucidal activity, we pre-incubated flavonoids with the virus suspension for 1h and then diluted the mixture 20-fold to sub-therapeutic concentrations before adding it to Vero cells. After 24h post-treatment, the virus was collected and quantified. Our results showed that 7,8-benzoflavone reduced the ASFV yield from 5.24±0.21 log TCID_50_/ml to 4.64±0.09 log TCID_50_/ml (*p*<0.05), indicating that this compound possesses virucidal activity (Figure 2C). However, no virucidal effects were observed for other flavonoids, including kaempferol. For cytotoxicity studies, Vero cells were treated with flavonoids in a dose-dependent manner. After 24h, cell viability was measured by a crystal violet staining method. We used this method instead of MTT because some flavonoids have been reported to interfere with mitochondrial function (Kicinska and Jarmuszkiewicz, 2020), which may lead to false-positive or -negative MTT results. None of the tested compounds caused cell monolayer damage or morphological changes at the screening concentration of 20μg/ml or 10μg/ml (for 7,8-benzoflavone, diosmin, and khellin). Furthermore, cell viability was higher than 70% for all flavonoids, except for isosinensetin (58.5%; Figure 2D). The CC_50_ values were 53.5μg/ml (196.5μm), 73.4μg/ml (258.3μm), 33.1μg/ml (54.4μm), 24.8μg/ml (66.6μm), 93.1μg/ml (325.3μm), 35.5μg/ml (136.4μm), 110.3μg/ml (388μm), 68.2μg/ml (238.3μm), and 150.5μg/ml (404.3μm) for 7,8-benzoflavone, calycosin, diosmin, isosinensetin, kaempferol, khellin, maackiain, sakuranetin, and sinensetin, respectively. Based on these results, we selected kaempferol for further investigation as it demonstrated the highest antiviral activity against ASFV among the tested flavonoids.

### Antiviral Effect of Kaempferol on ASFV Infection in Vero Cells

**Figure 3:**
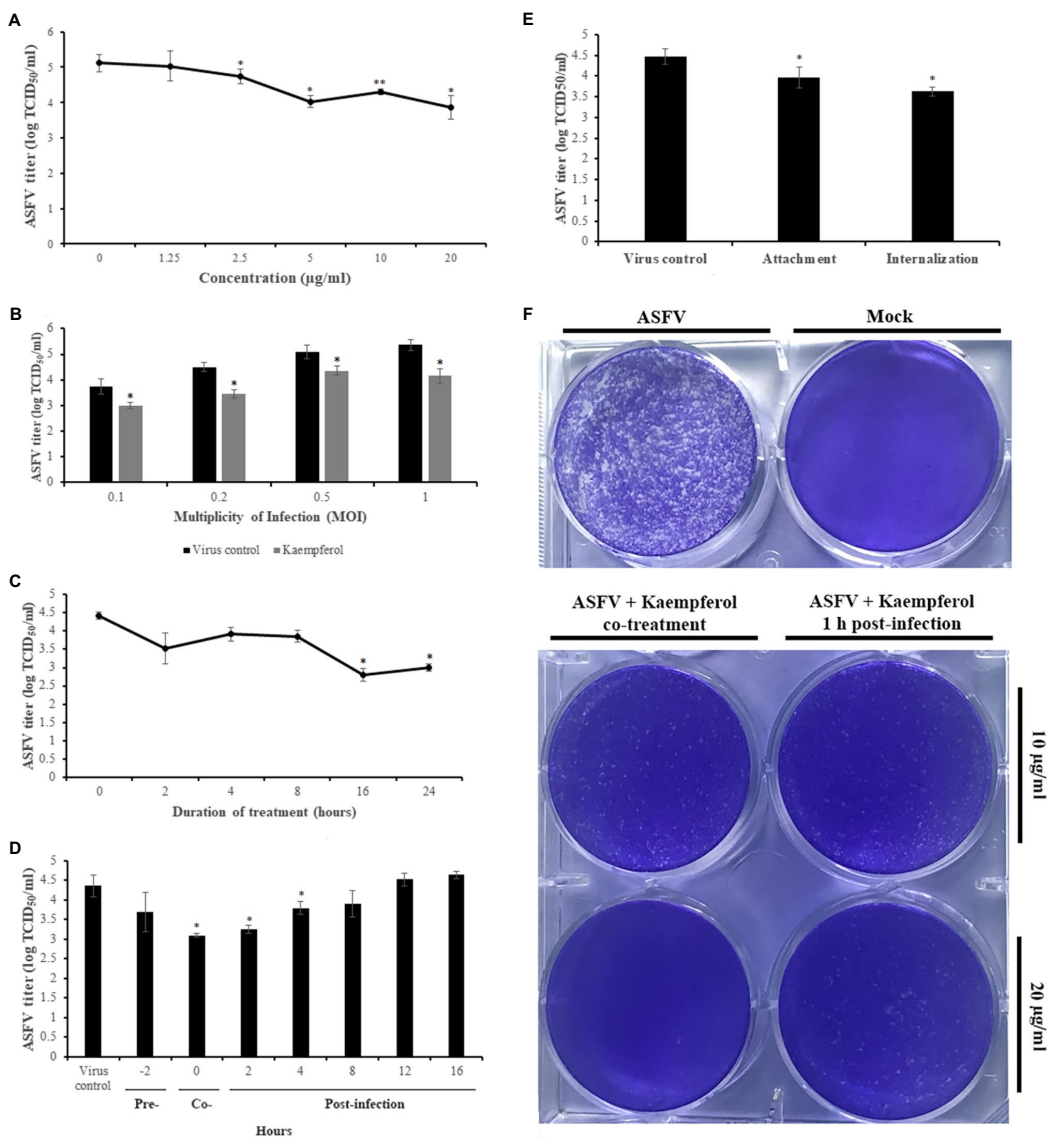
Antiviral activity of Kaempferol against ASFV in Vero cells. **(A)** ASFV titer in cells exposed to kaempferol at increasing concentrations from 1.25μg/ml (4.3μm) to 20μg/ml (70μm). **(B)** Effect of kaempferol on Vero cells infected with ASFV at different MOIs. **(C)** Time-of-removal effect of kaempferol on ASFV-infected cells to determine the optimal treatment time. **(D)** Antiviral activity of kaempferol depending on the time of addition. **(E)** Effect of kaempferol treatment on ASFV entry stage. **(F)** ASFV spread in cells treated with kaempferol. The concentration of tested compound on in the experiments in panels **(B)**, **(C)**, **(D)**, and **(E)** was 20μg/ml. Results represent the mean (± SD) of three independent experiments (*n*=3). Significant differences compared to control are denoted by ^*^p<0.05.

To determine the effects of kaempferol concentration on antiviral activity against ASFV, we treated ASFV-infected Vero cells with kaempferol at different compound concentrations. As shown in Figure 3A, the strongest antiviral effect, a 1.26 log decrease, was recorded at 20μg/ml (70μm) concentration. This inhibitory effect occurred in a dose-dependent manner down to around 2.5μg/ml. Based on the cytotoxicity and dose-dependent antiviral experiments (*cf*. Figures 2D, 3A), we calculated the IC_50_ and SI of kaempferol, which were 2.2μg/ml (7.7μm) and 42.3, respectively. Furthermore, treatment of ASFV-infected Vero cells with 20μg/ml kaempferol resulted in ASFV inhibition at all tested MOIs with the strongest inhibition (1.21 log decrease) at an MOI of 1 (Figure 3B). Since kaempferol demonstrated the strongest inhibitory effect at 20μg/ml among the tested concentrations, we decided to perform further studies using this concentration.

Next, we determined the optimal treatment duration required for the strongest antiviral effect on ASFV replication. In these experiments, kaempferol was added at 0h post-infection and was then removed through medium replacement after specific intervals. After 24h post-infection, the virus was collected and quantified. As shown in Figure 3C, gradual declines in viral titers were observed as the interval between infection and kaempferol removal increased.

To determine the specific replication stages in the ASFV life cycle that can be affected by kaempferol, we conducted time-of-addition experiments. Kaempferol was added at seven time points prior to and after infection followed by virus titration at 24h post-infection (Figure 3D). In these time-of-addition experiments, a sharp drop in virus titer was observed at the early stages of infection. Notably, the most potent antiviral effects were observed when kaempferol was added at 0 and 2h post-infection, which led to viral titer reductions of 1.25 log (*p*<0.05) and 1.1 log (*p*<0.05), respectively. In marked contrast, there was no significant inhibitory effect on ASFV infection when kaempferol was added at later stages of infection.

Since kaempferol affected early stages of ASFV infection, we further studied the antiviral activity of kaempferol when it was added at the virus entry stage (i.e., during attachment and internalization). To determine whether kaempferol interferes with ASFV attachment to Vero cells, it was added at 4°C, a condition in which the virus binds to but does not enter cells. As shown in Figure 3E, treatment with kaempferol reduced the viral titer from 4.48±0.19 log TCID_50_/ml to 3.97±0.25 log TCID_50_/ml (*p*<0.05), resulting in about 60% inhibition of the ASFV yield. We also studied the effect of kaempferol on the ASFV internalization stage. For this purpose, the compound was added immediately after a temperature shift from 4°C to 37°C and removed after 1h of treatment, to minimize potential further antiviral effects on subsequent stages of ASFV replication. In this case, the virus titer decreased from 4.48±0.19 log TCID_50_/ml to 3.63±0.11 log TCID_50_/ml (*p*<0.05; Figure 3E).

In addition, we conducted a liquid plaque assay to define whether kaempferol can inhibit virus spread *in vitro*. Kaempferol was added to Vero cells together with ASFV (0h) or 1h post-infection. The results showed a significant reduction in plaque numbers in a dose-dependent manner, indicating that the viral spread was diminished in the presence of kaempferol (Figure 3F).

### Inhibitory Effect on ASFV Protein and DNA Synthesis

**Figure 4:**
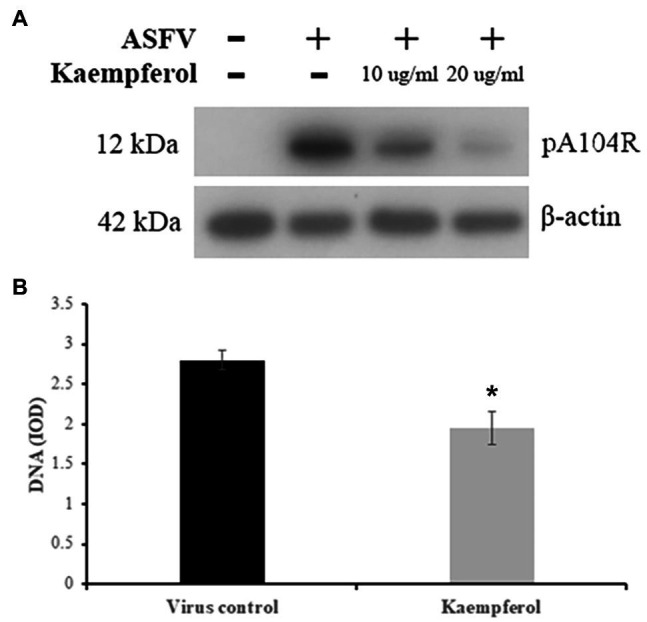
Inhibition of ASFV protein and DNA synthesis in Vero cells. **(A)** Western blot analysis of ASFV late protein in ASFV-infected cells exposed to kaempferol at different concentrations. β-actin was used as a loading control. **(B)** DNA content of ASFV factories in cells treated with kaempferol. Results represent the mean (± SD) of three independent experiments (*n*=3). Significant differences compared to control are denoted by ^*^p<0.05.

Since our findings showed that kaempferol disrupts ASFV replication, we further investigated the effects of kaempferol on ASFV protein synthesis by evaluating the expression of a late ASFV protein (pA104R) through western blot analysis (Frouco et al., 2017). The results showed that viral protein synthesis was reduced following treatment with 20 (70μm) or 10μg/ml (35μm) kaempferol at an MOI of 5 in a dose-dependent manner (Figure 4A).

To determine whether kaempferol may affect viral DNA replication, we quantified ASFV DNA in viral factories by performing single-cell image cytometry developed for ASFV (Karalyan et al., 2018). As shown in Figure 4B, the amount of viral DNA was significantly less than in the untreated control by 31% (*p*<0.05), when ASFV-infected Vero cells were exposed to kaempferol from 1h post-infection onwards, suggesting that viral DNA synthesis was impaired in the presence of kaempferol.

### Inhibition of ASFV Infection in Porcine Macrophages

**Figure 5:**
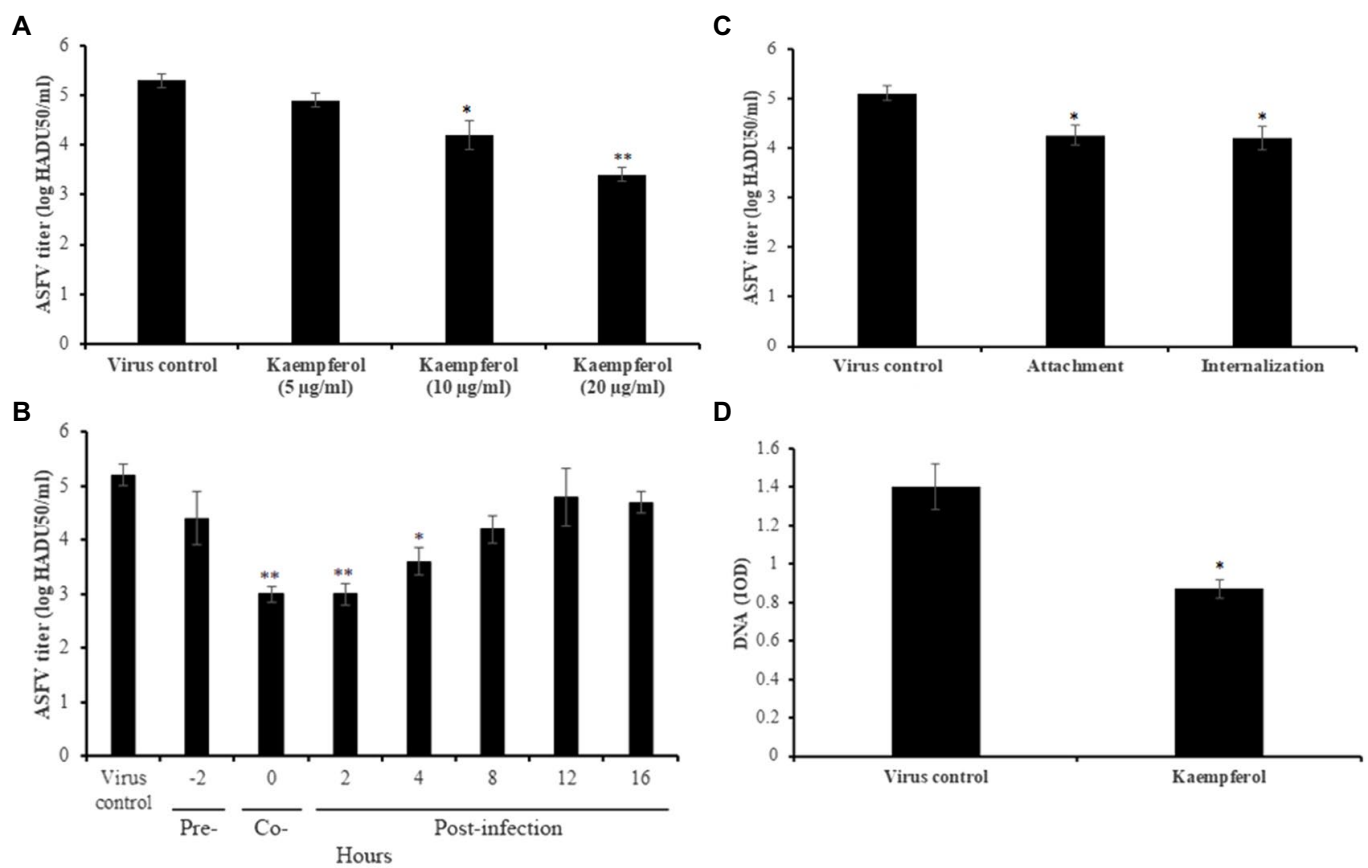
Antiviral activity of kaempferol against ASFV Arm/07 isolate in porcine macrophages. **(A)** ASFV titer in macrophages treated with kaempferol at increasing concentrations from 5μg/ml (17.5μm) to 20μg/ml (70μm). **(B)** Effect of kaempferol on different stages of ASFV infection in macrophages. **(C)** Effect of kaempferol on ASFV entry into macrophages. **(D)** DNA content of ASFV factories in macrophages treated with 20μg/ml kaempferol. Results represent the mean (± SD) of three independent experiments (*n*=3). Significant differences compared to control are denoted by ^*^p<0.05 and ^**^p<0.01.

Since monocytes and macrophages are the main cells for ASFV replication in pigs, we studied the antiviral activity of kaempferol against a highly virulent ASFV isolate (Arm/07) in porcine alveolar macrophages. For this purpose, ASFV-infected macrophages were treated with kaempferol at increasing concentrations up to 20μg/ml, which was not cytotoxic for porcine cells (data not shown). Anti-ASFV activity was observed at 10 and 20μg/ml concentrations in a dose-dependent manner (Figure 5A). Kaempferol at 20μg/ml concentration reduced the viral titer from 5.3±0.11 log HADU_50_/ml to 3.4±0.14 log HADU_50_/ml (*p*<0.01), thereby demonstrating more potent antiviral activity than in Vero cells. The most potent inhibition (about 2 log decrease) was observed, when kaempferol was added at very early stages of infection, which was in agreement with results in Vero cells (Figure 5B). Anti-entry experiments also demonstrated that kaempferol could hinder the ASFV attachment and internalization processes (Figure 5C). Finally, a significant decrease in the amount of viral DNA in ASFV factories was observed (39%, *p*<0.05), when ASFV-infected macrophages were exposed to kaempferol from 1h post-infection onwards (Figure 5D). Thus, these results indicate that the inhibitory effect of kaempferol against ASFV is agnostic to the virus strain and host cells.

### Interplay Between Autophagy and Antiviral Activity of Kaempferol

**Figure 6:**
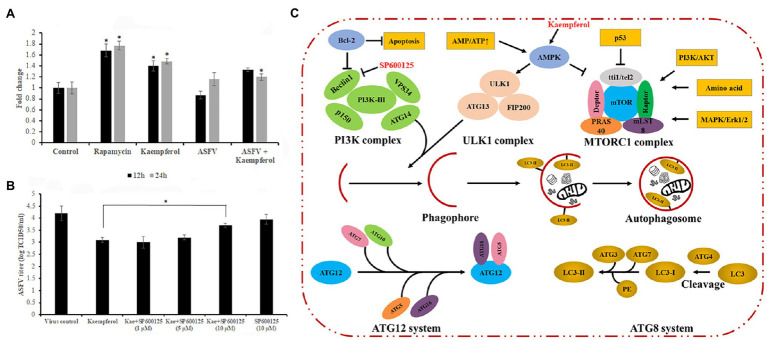
Role of autophagy in the antiviral effect of kaempferol. **(A)** Effect of kaempferol on the formation of autophagic vacuoles in Vero cells. **(B)** Reverse effect of autophagy inhibitor SP600125 on the antiviral activity of kaempferol in ASFV-infected Vero cells. **(C)** Regulation of autophagy signaling pathway. Kaempferol causes AMPK activation, which can lead to autophagy induction through downstream signaling. These signaling pathways eventually result in phagophore formation and subsequent stages involve autophagosome maturation, lysosomal fusion, and finally cargo degradation. The SP600125 inhibitor can prevent Beclin-1 upregulation as part of repressing the JNK-Beclin1 signaling pathway, which can in turn inhibit autophagy induction (Parzych and Klionsky, 2014). Results in **(A)** and **(B)** graphs represent the mean (± SD) of three independent experiments (*n*=3). Significant differences compared to control are denoted by ^*^p<0.05.

Past studies have reported that kaempferol may induce autophagy in various cell lines (Huang et al., 2013; Varshney et al., 2017; Hoang et al., 2019). On the other hand, it has been shown that autophagy induction hampers ASFV infection *in vitro* (Hernaez et al., 2013). We hypothesized that the antiviral activity of kaempferol at the post-entry stage of ASFV replication may be attributed to autophagy induction. To test this hypothesis, mock and ASFV-infected Vero cells were treated with kaempferol and the level of monodansylcadaverine, a fluorescent marker of autophagic vacuoles, was measured at 12 and 24h post-treatment and compared with control values. As shown in Figure 6A and Supplementary Table S2, kaempferol induced the formation of autophagic vacuoles in mock and ASFV-infected Vero cells at both time points. Then, ASFV-infected cells were exposed to kaempferol in the presence of an autophagy inhibitor, SP600125, at increasing inhibitor concentrations. The antiviral activity of kaempferol was partially abrogated by the addition of the autophagy inhibitor, thus confirming autophagy induction as an antiviral mechanism against ASFV (Figure 6B).

## Discussion

The first efforts to develop vaccines against ASFV were initiated in the 1960s, when Stone and Hess demonstrated that inactivated ASFV is not a feasible vaccine strategy (Stone and Hess, 1967). Since those days, various vaccine candidates, such as conventional attenuated vaccines, gene-deleted vaccines, and DNA vaccines, have been tested with limited success (Zakaryan and Revilla, 2016; Arias et al., 2017; Bosch-Camós et al., 2020). The genetic diversity among ASFV genotypes and immune modulation strategies implemented by the virus challenge the development of a safe and efficacious ASFV vaccine (Reis et al., 2017; Malogolovkin and Kolbasov, 2019). Due to the lack of effective vaccines, the development of antiviral strategies to prevent and control ASFV outbreaks is a high priority.

Nucleoside analogues, interferons and their derivatives, antibiotics, and siRNAs have been shown to effectively inhibit ASFV replication in cell lines and in porcine macrophages (Gil-Fernandez et al., 1979, 1987; Paez et al., 1990; Keita et al., 2010; Mottola et al., 2013; Freitas et al., 2016). However, these compounds have little chance to become commercially successful anti-ASFV drugs as most of them are expensive or unsafe. Therefore, we have focused our attention on the anti-ASFV activity of natural compounds because they are less toxic, cheaper, and can be used, for example, as feed additives in pig nutrition (Jackman et al., 2020). Previously, we have demonstrated the antiviral properties of several flavonoids against ASFV *in vitro* (Hakobyan et al., 2016, 2019; Arabyan et al., 2018), thereby justifying our efforts to screen more flavonoids.

In this study, we used a cell-based screening platform to screen a library of 90 flavonoids for inhibitory activity against ASFV. Our screening platform was shown to be robust for hit identification, with an average Z-factor of 0.683 for the entire screening process that supports the assay has suitable dynamic range and low data variability (Zhang et al., 1999). The screening process identified nine flavonoids with ≥40% inhibition of ASFV-induced CPE in Vero cells. The antiviral activity of all nine compounds was further characterized by virostatic, virucidal, and cytotoxicity experiments. Among these flavonoids, kaempferol displayed the strongest inhibition of ASFV replication at non-cytotoxic concentrations and therefore was chosen as the lead compound for detailed mechanistic studies.

Kaempferol [3,5,7-trihydroxy-2-(4-hydroxyphenyl)-4*H*-chromen-4-one] is a polyphenol that is found in various fruits and vegetables, such as beans, spinach, and broccoli, as well as in plant-based beverages. Kaempferol is a lipophilic molecule; hence, it is readily absorbed from the small intestine (DuPont et al., 2004). Bioavailability studies have shown that its human plasma concentration was around 58nm when a 15mg/day kaempferol dose was given daily (de Vries et al., 1998; DuPont et al., 2004). While the oral bioavailability of kaempferol is relatively low, additional studies have validated its therapeutic potential and kaempferol inhibits cyclooxygenase enzymes and exhibits anti-inflammatory properties *in vitro*, *in vivo*, and in humans (García-Mediavilla et al., 2007; Kong et al., 2013; Hosseinpour-Niazi et al., 2015). Kaempferol has also been shown to have antiviral activity against several viruses such as herpes simplex virus 1, Japanese encephalitis virus, and influenza B virus (Lyu et al., 2005; Zhang et al., 2012; Yang et al., 2014).

In this study, we showed that kaempferol was effective at inhibiting ASFV infection in a permissible Vero cell line and in porcine macrophages. We also found that kaempferol affected virus replication at two distinct stages. Kaempferol inhibited the ASFV yield by more than 60%, when it was added at the virus entry stage. The lack of inhibitory effect when ASFV was incubated with kaempferol in the virucidal assay indicates that the anti-entry activity was not associated with the direct effect of the compound on ASFV particles. To our knowledge, this is the first study displaying that kaempferol could interfere with the viral entry process. Although the mechanisms by which kaempferol interferes with ASFV entry remain to be elucidated, we hypothesize that the anti-entry activity is more ASFV-specific rather than broad-spectrum phenomenon because it has not been observed in other studies. Besides its anti-entry activity, kaempferol also displayed potent anti-ASFV activity at a post-entry stage, suppressing viral infection by more than 90% when it was added at early phases of infection (0 to 4h post-infection). It is well established that kaempferol may induce autophagy in different cell lines through the upregulation of p-AMP-activated protein kinase (Huang et al., 2013; Varshney et al., 2017; Hoang et al., 2019). Autophagy is a self-degradation process that is conserved among eukaryotic cells. Under different conditions, such as starvation, misfolded proteins, or invading pathogens, autophagy is activated through a signaling process that effectively degrades these substrates (Figure 6C). Depending on the type of virus, autophagy can either benefit or hinder virus replication. For instance, hepatitis B virus and dengue virus facilitate their replication and maturation by enhancing autophagy (Jordan and Randall, 2017; Lin et al., 2020). In contrast, autophagy induction by starvation or pharmacological inhibitors blocks ASFV infection *in vitro* (Hernaez et al., 2013). Here, we hypothesized that the anti-ASFV activity of kaempferol at the post-entry stage could be associated with its autophagy induction properties in ASFV-infected cells. Indeed, our results showed a significant increase in the number of autophagic vacuoles in Vero cells upon treatment with kaempferol. Then, to determine whether autophagy was involved in the inhibition of ASFV infection, the autophagy inhibitor SP600125 was added to ASFV-infected cells exposed to kaempferol. Since most autophagy inhibitors act on PI3K or endosomal acidification levels that are essential for ASFV replication and therefore they may have additional therapeutic effects on ASFV, we used SP600125 that blocks autophagy by repressing the JNK-Beclin1 signaling pathway (Li et al., 2009). As expected, the viral titers were partially restored with the addition of SP600125, whereas the inhibitor alone had only a slight effect on ASFV infection. These findings support our hypothesis about the role of autophagy in the anti-ASFV activity of kaempferol.

## Conclusion

In conclusion, we have established a robust cell-based library screening platform, which was employed to screen a library of 90 flavonoids. Kaempferol was identified as a hit and was shown to display potent dose-dependent inhibition against ASFV in Vero cells and in porcine macrophages. Kaempferol was shown to act on the entry and post-entry stages of the ASFV replication cycle. While the exact mechanism of action of kaempferol on ASFV entry still remains to be studied, we demonstrated that kaempferol-induced autophagy was involved in virus inhibition at the post-entry stage. Since the results of this study were based on *in vitro* experiments, future *in vivo* studies would be advantageous to further assess the efficacy of kaempferol in ASFV-infected pigs.

## Data Availability Statement

The original contributions presented in the study are included in the article/Supplementary Material, further inquiries can be directed to the corresponding authors.

## Author Contributions

FF, CE, and HZ conceived and designed the experiments. EA, AH, TH, RG, RI, AA, and ZK performed the experiments. JJ, FF, CE, and HZ analyzed the data and wrote the manuscript. All authors approved the submitted manuscript.

## Funding

This work was partially supported by the Science Committee of the Ministry of Education, Science, Culture and Sport RA (grant number: 19YR-1F039) and by Fundação para a Ciência e a Tecnologia (Portugal), through the project UIDB/00276/2020.

## Conflict of Interest

CE was employed by company Natural Biologics Inc.

The remaining authors declare that the research was conducted in the absence of any commercial or financial relationships that could be construed as a potential conflict of interest.

## Publisher’s Note

All claims expressed in this article are solely those of the authors and do not necessarily represent those of their affiliated organizations, or those of the publisher, the editors and the reviewers. Any product that may be evaluated in this article, or claim that may be made by its manufacturer, is not guaranteed or endorsed by the publisher.

## Abbreviations

ASFV: African swine fever virus
CPE: cytopathic effect
TCID: issue culture infective dose
HADU: hemadsorption unit
DMSO: dimethyl sulfoxide
MTT: 3-(4,5-dimethylthiazol-2-yl)-2,5-diphenyltetrazolium bromide
PBS: phosphate-buffered saline
OD: optical density
MOI: multiplicity of infection
PFU: plaque-forming unit
PBST: phosphate-buffered saline with tween
HRP: horseradish peroxidase
