# Supplementary material for: Flavonoid Library Screening Reveals Kaempferol as a Potential Antiviral Agent Against African Swine Fever Virus
Source: Front Microbiol. 2021 Oct 21;12:736780. doi: 10.3389/fmicb.2021.736780 (PMC8567988; doi:10.3389/fmicb.2021.736780)
Supplement: Supplementary file 1 [file Table_1.DOCX]

| \|  \| Compound \| Structure \| Molecular weight \| \| --- \| --- \| --- \| --- \| \| 1 \| Maltol \| 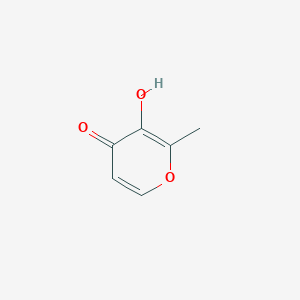 \| 126.11g/mol \| \| 2 \| 4-hydroxycoumarin \| 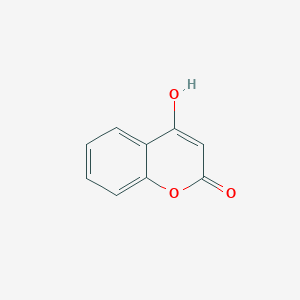 \| 162.14g/mol \| \| 3 \| Formononetin \| 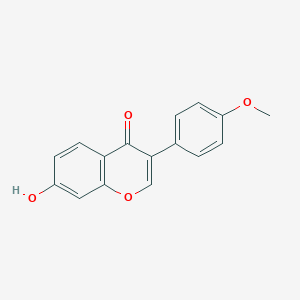 \| 268.26g/mol \| \| 4 \| Morin \| 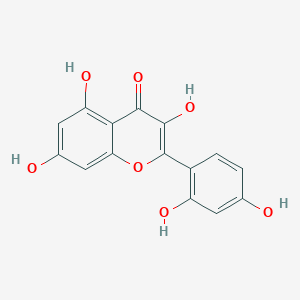 \| 302.23g/mol \| \| 5 \| Myricitrin \| 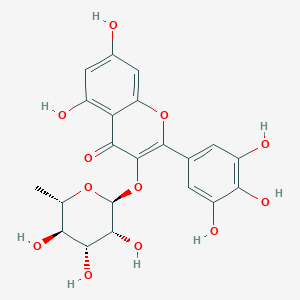 \| 464.4g/mol \| \| 6 \| Lysionotin \| 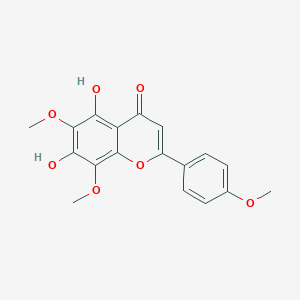 \| 344.3g/mol \| \| 7 \| Methyl hesperidin \| 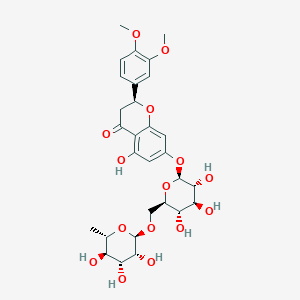 \| 624.6 g/mol \| \| 8 \| Epicatechin gallate \| 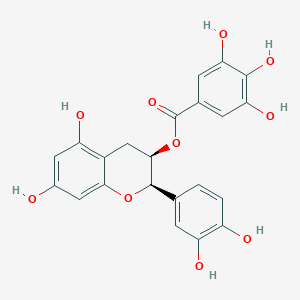 \| 442.4g/mol \| \| 9 \| Epigallocatechin gallate \| 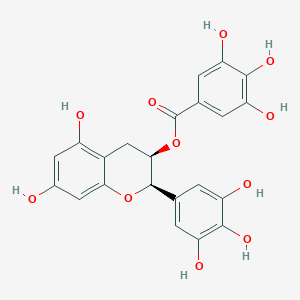 \| 458.4g/mol \| \| 10 \| Naringin \| 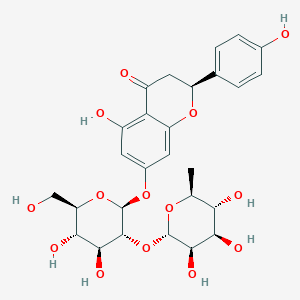 \| 580.5g/mol \| \| 11 \| Icariin \| 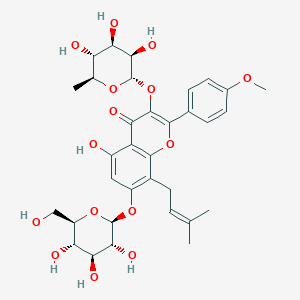 \| 676.7g/mol \| \| 12 \| Silymarin \| 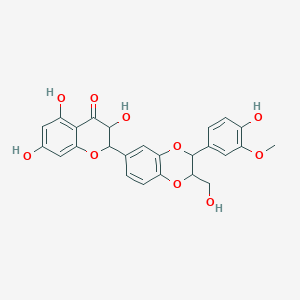 \| 482.4g/mol \| \| 13 \| Puerarin \| 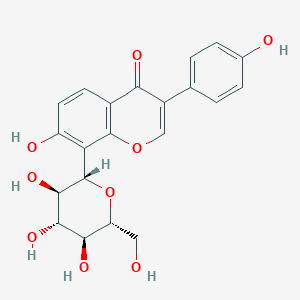 \| 416.4g/mol \| \| 14 \| Scutellarin \| 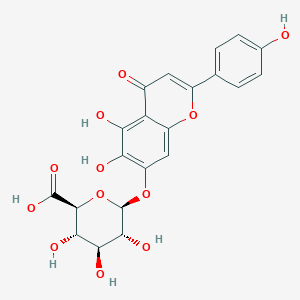 \| 462.4 g/mol \| \| 15 \| Baicalin \| 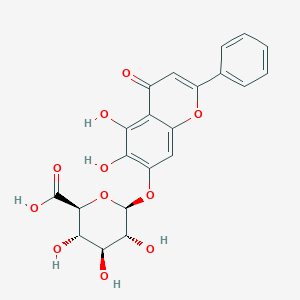 \| 446.4g/mol \| \| 16 \| Daidzin \| 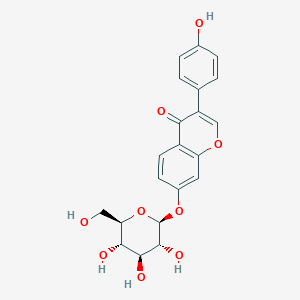 \| 416.4g/mol \| \| 17 \| Apigenin \| 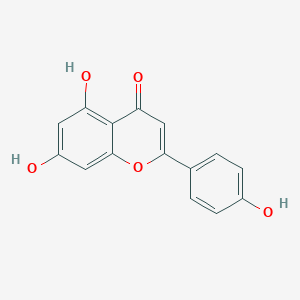 \| 270.24g/mol \| \| 18 \| Hesperetin \| 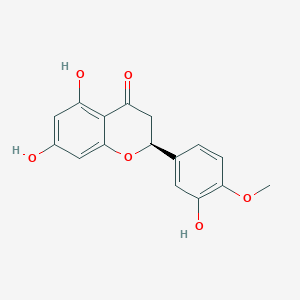 \| 302.28g/mol \| \| 19 \| Hesperidin \| 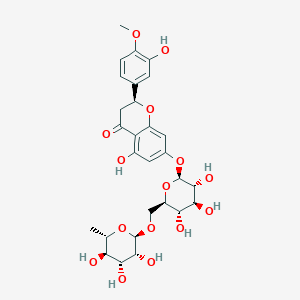 \| 610.6g/mol \| \| 20 \| Kaempferol \| 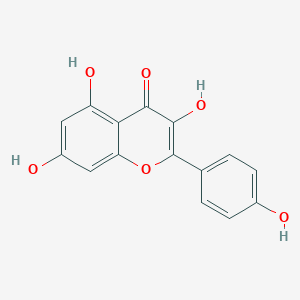 \| 286.24g/mol \| \| 21 \| Baicalein \| 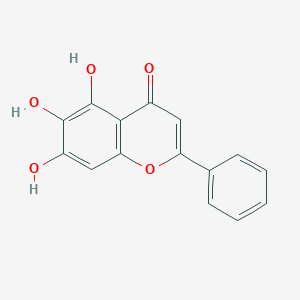 \| 270.24g/mol \| \| 22 \| Daidzein \| 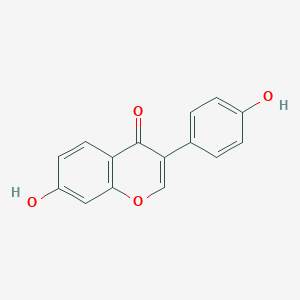 \| 254.24 g/mol \| \| 23 \| Hyperoside \| 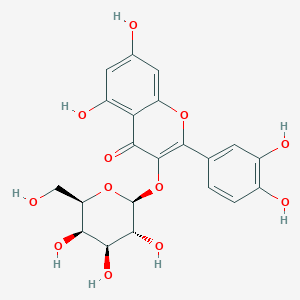 \| 464.4g/mol \| \| 24 \| Chrysin \| 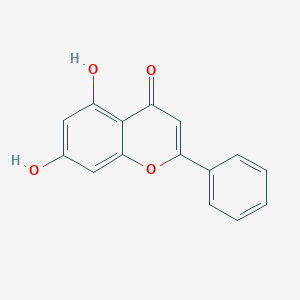 \| 254.24g/mol \| \| 25 \| Taxifolin \| 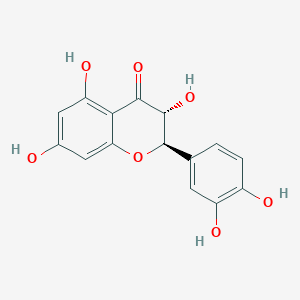 \| 304.25g/mol \| \| 26 \| Nobiletin \| 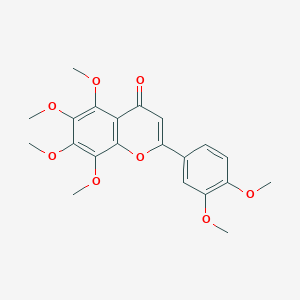 \| 402.4g/mol \| \| 27 \| Ampelopsin \| 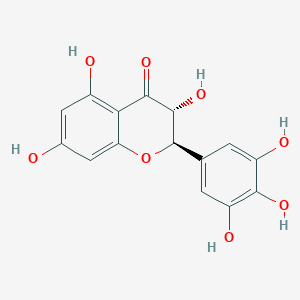 \| 320.25 g/mol \| \| 28 \| Fisetin \| 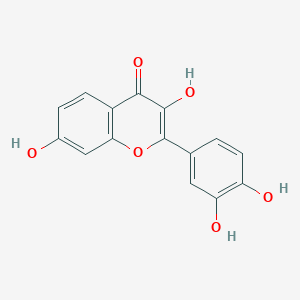 \| 286.24g/mol \| \| 29 \| Diosmin \| 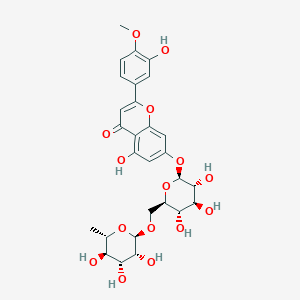 \| 608.5g/mol \| \| 30 \| Khellin \| 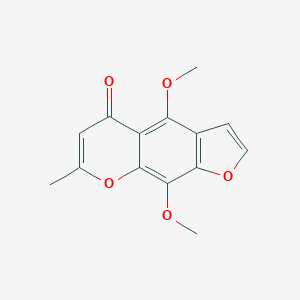 \| 260.24g/mol \| \| 31 \| Nevadensin \| 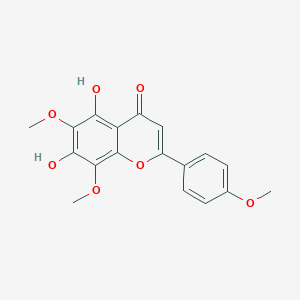 \| 344.3 g/mol \| \| 32 \| 7,8-benzoflavone \| 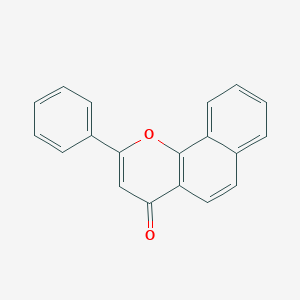 \| 272.3 g/mol \| \| 33 \| Genistin \| 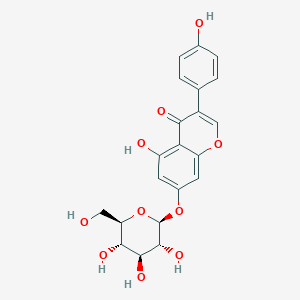 \| 432.4 g/mol \| \| 34 \| Wogonin \| 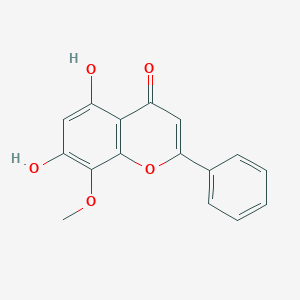 \| 284.26 g/mol \| \| 35 \| Catechin hydrate \| 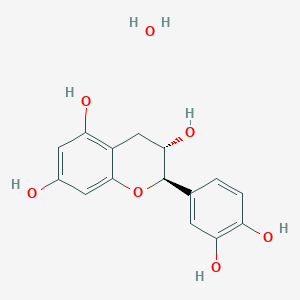 \| 308.28g/mol \| \| 36 \| Amentoflavone \| 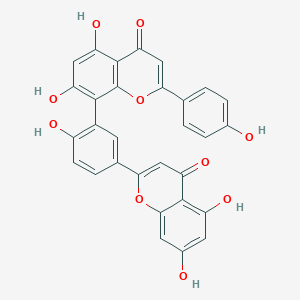 \| 538.5 g/mol \| \| 37 \| Liquiritin \| 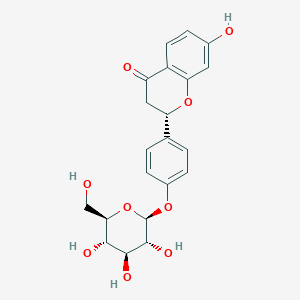 \| 418.4 g/mol \| \| 38 \| Glycitin \| 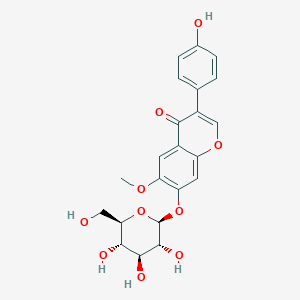 \| 446.4 g/mol \| \| 39 \| Quercetrin \| 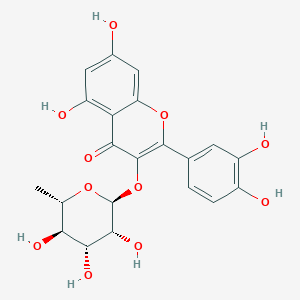 \| 448.4 g/mol \| \| 40 \| Pinocembrin \| 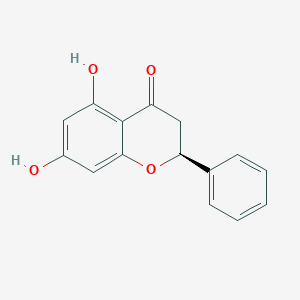 \| 256.25 g/mol \| \| 41 \| Epigallocatechin \| 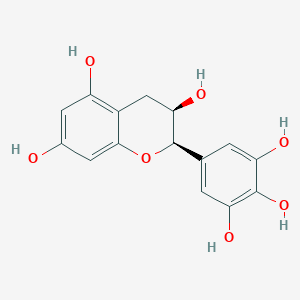 \| 306.27 g/mol \| \| 42 \| Visnagin \| 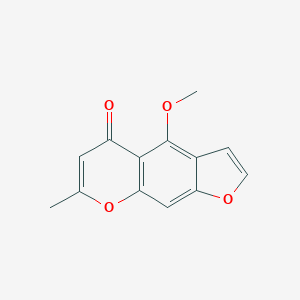 \| 230.22 g/mol \| \| 43 \| Tangeretin \| 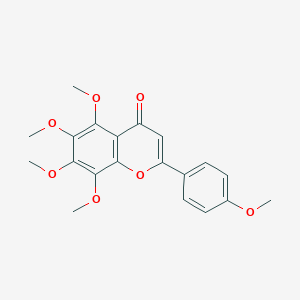 \| 372.4 g/mol \| \| 44 \| Glabridin \| 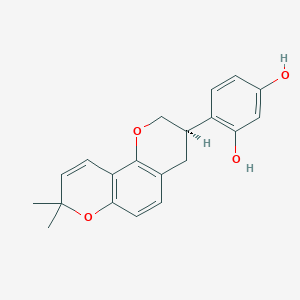 \| 324.4 g/mol \| \| 45 \| Gallocatechin gallate \| 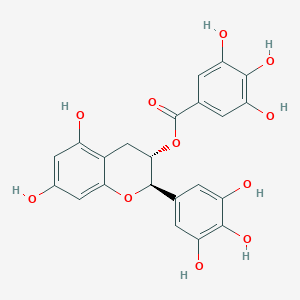 \| 458.4 g/mol \| \| 46 \| Liquiritigenin \| 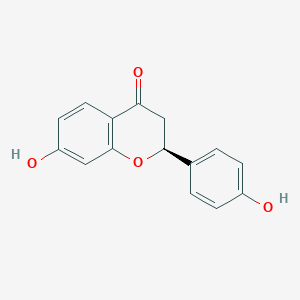 \| 256.25 g/mol \| \| 47 \| Galangin \| 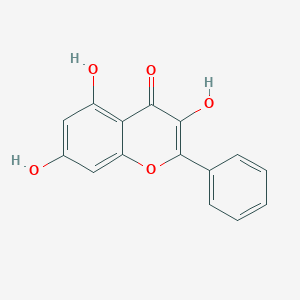 \| 270.24 g/mol \| \| 48 \| Myricetin \| 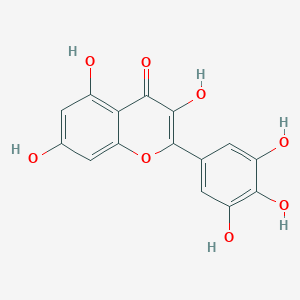 \| 318.23 g/mol \| \| 49 \| Kaempferide \| 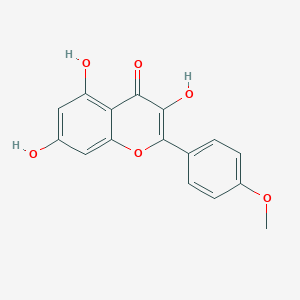 \| 300.26 g/mol \| \| 50 \| Isoquercitrin \| 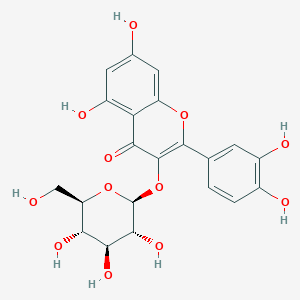 \| 464.4 g/mol \| \| 51 \| Isorhamnetin \| 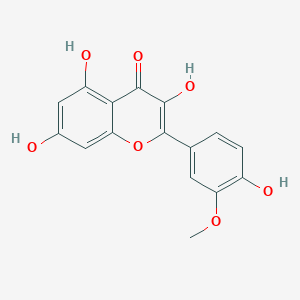 \| 316.26 g/mol \| \| 52 \| Astilbin \| 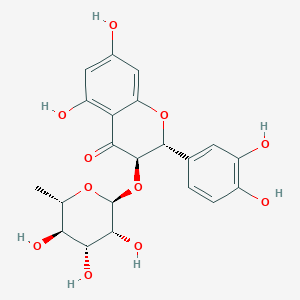 \| 450.4g/mol \| \| 53 \| Chrysin dimethylether \| 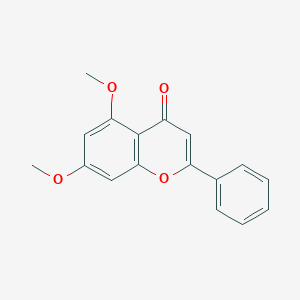 \| 282.29 g/mol \| \| 54 \| Biochanin A \| 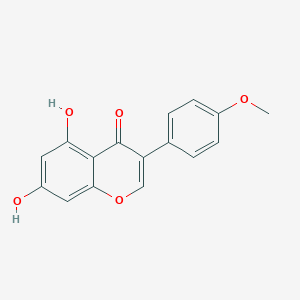 \| 284.26 g/mol \| \| 55 \| Calycosin \| 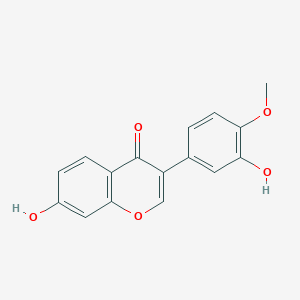 \| 284.26 g/mol \| \| 56 \| Cimifugin \| 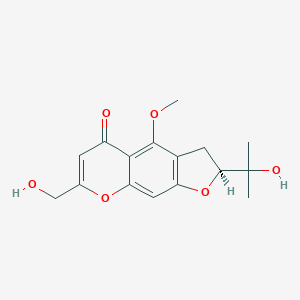 \| 306.31 g/mol \| \| 57 \| Scutellarein \| 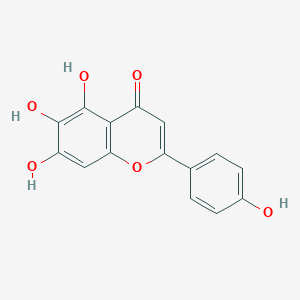 \| 286.24 g/mol \| \| 58 \| Icaritin \| 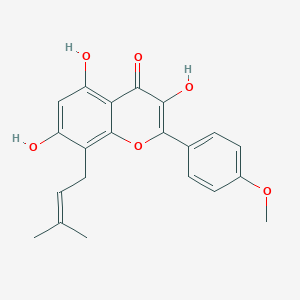 \| 368.4 g/mol \| \| 59 \| Narirutin \| 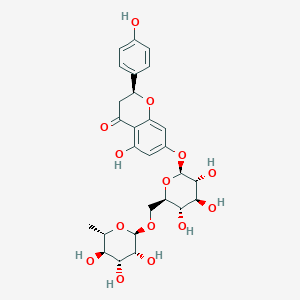 \| 580.5 g/mol \| \| 60 \| Kaempferitrin \| 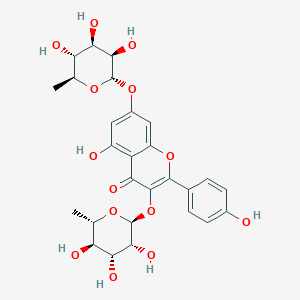 \| 578.5 g/mol \| \| 61 \| Irisflorentin \| 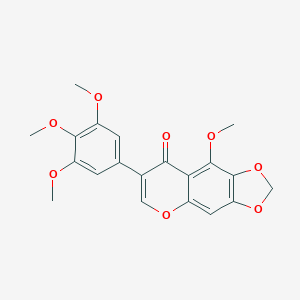 \| 386.4 g/mol \| \| 62 \| Noricaritin \| 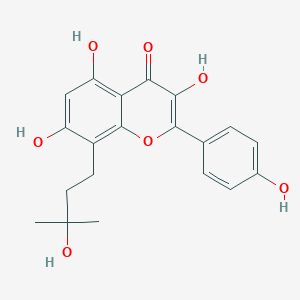 \| 372.4 g/mol \| \| 63 \| Glycitein \| 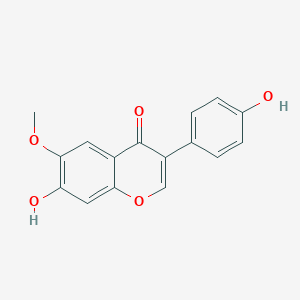 \| 284.26 g/mol \| \| 64 \| Corylin \| 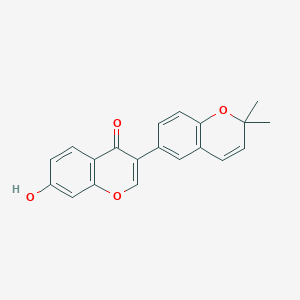 \| 320.3 g/mol \| \| 65 \| Morusin \| 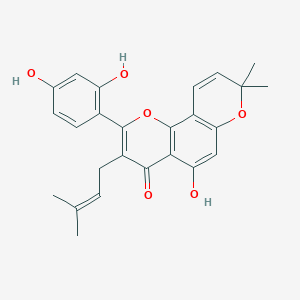 \| 420.5 g/mol \| \| 66 \| Didymin \| 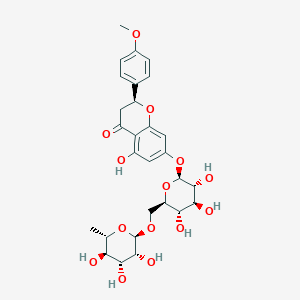 \| 594.6 g/mol \| \| 67 \| Neobavaisoflavone \| 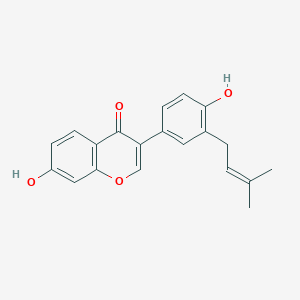 \| 322.4 g/mol \| \| 68 \| Tectorigenin \| 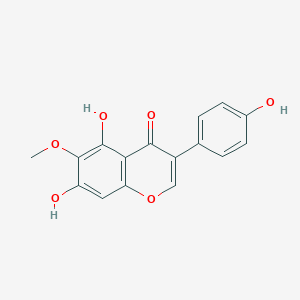 \| 300.26 g/mol \| \| 69 \| Cosmosiin \| 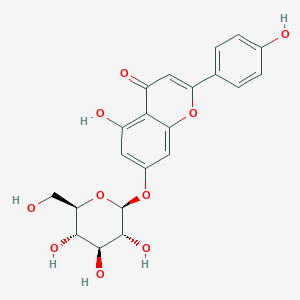 \| 432.4 g/mol \| \| 70 \| Astragalin \| 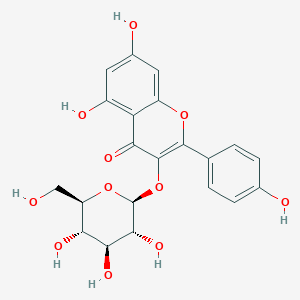 \| 448.4 g/mol \| \| 71 \| Oroxylin A \| 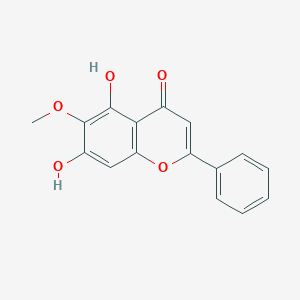 \| 284.26 g/mol \| \| 72 \| Orientin \| 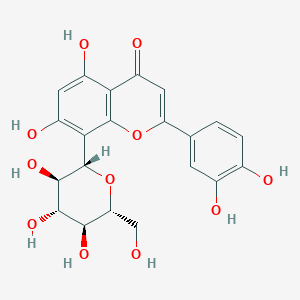 \| 448.4 g/mol \| \| 73 \| Sinensetin \| 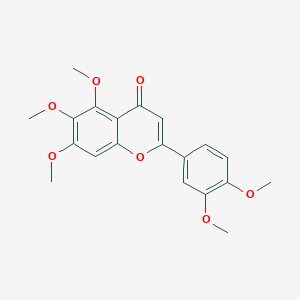 \| 372.4 g/mol \| \| 74 \| Ononin \| 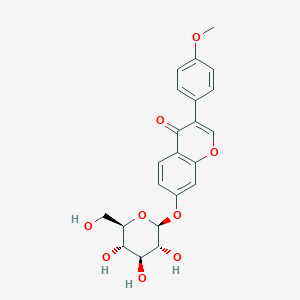 \| 430.4 g/mol \| \| 75 \| Engeletin \| 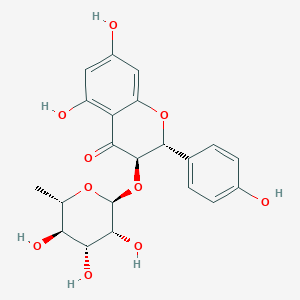 \| 434.4 g/mol \| \| 76 \| Brazilin \| 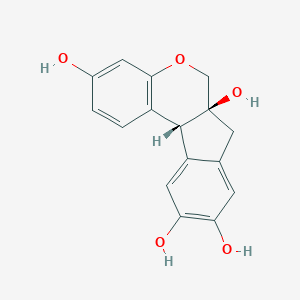 \| 286.28 g/mol \| \| 77 \| Farrerol \| 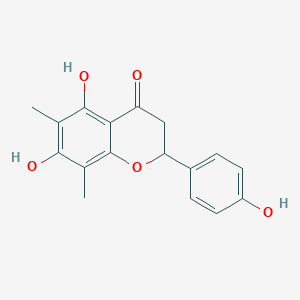 \| 300.3 g/mol \| \| 78 \| Pectolinarigenin \| 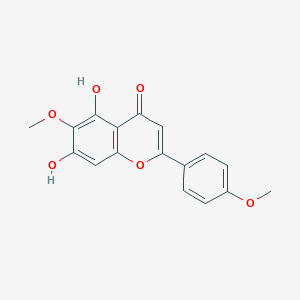 \| 314.29 g/mol \| \| 79 \| Isobavachin \| 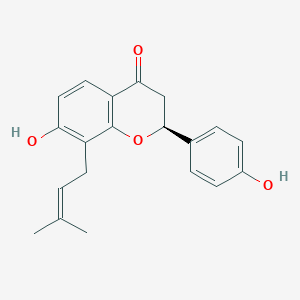 \| 324.4 g/mol \| \| 80 \| Mosloflavone \| 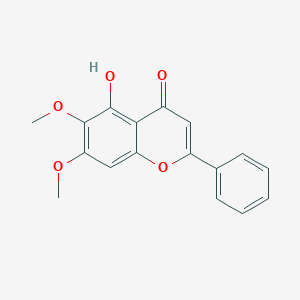 \| 298.29 g/mol \| \| 81 \| Maackiain \| 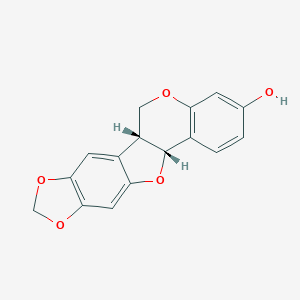 \| 284.26 g/mol \| \| 82 \| Sakuranetin \| 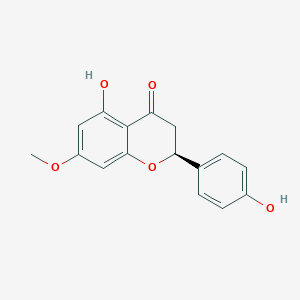 \| 286.28 g/mol \| \| 83 \| Gallocatechin \| 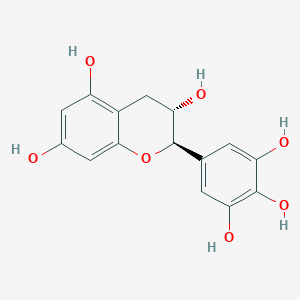 \| 306.27 g/mol \| \| 84 \| Isosakuranetin \| 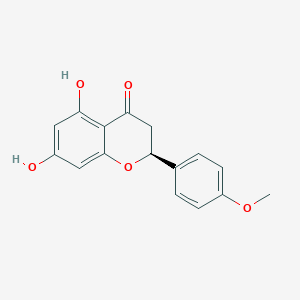 \| 286.28 g/mol \| \| 85 \| Isosinensetin \| 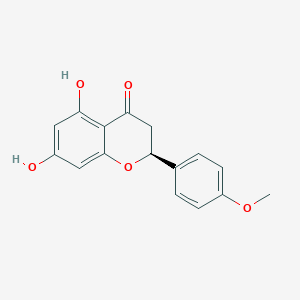 \| 286.28 g/mol \| \| 86 \| Catechin gallate \| 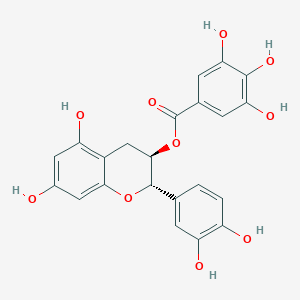 \| 442.4 g/mol \| \| 87 \| Pinostrobin \| 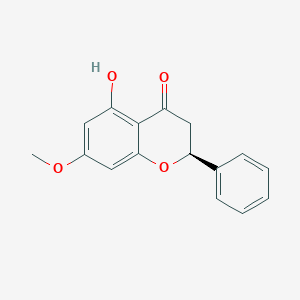 \| 270.28 g/mol \| \| 88 \| Silychristin \| 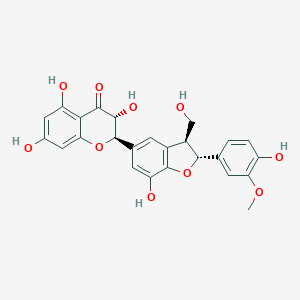 \| 482.4 g/mol \| \| 89 \| Butin \| 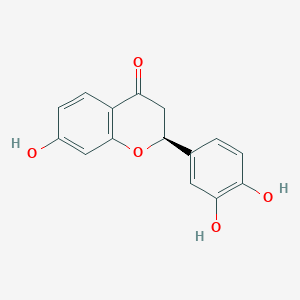 \| 272.25 g/mol \| \| 90 \| (+)-taxifolin \| 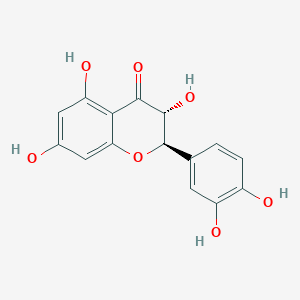 \| 304.25g/mol \| | |  |
| --- | --- | --- | --- | --- | --- | --- | --- | --- | --- | --- | --- | --- | --- | --- | --- | --- | --- | --- | --- | --- | --- | --- | --- | --- | --- | --- | --- | --- | --- | --- | --- | --- | --- | --- | --- | --- | --- | --- | --- | --- | --- | --- | --- | --- | --- | --- | --- | --- | --- | --- | --- | --- | --- | --- | --- | --- | --- | --- | --- | --- | --- | --- | --- | --- | --- | --- | --- | --- | --- | --- | --- | --- | --- | --- | --- | --- | --- | --- | --- | --- | --- | --- | --- | --- | --- | --- | --- | --- | --- | --- | --- | --- | --- | --- | --- | --- | --- | --- | --- | --- | --- | --- | --- | --- | --- | --- | --- | --- | --- | --- | --- | --- | --- | --- | --- | --- | --- | --- | --- | --- | --- | --- | --- | --- | --- | --- | --- | --- | --- | --- | --- | --- | --- | --- | --- | --- | --- | --- | --- | --- | --- | --- | --- | --- | --- | --- | --- | --- | --- | --- | --- | --- | --- | --- | --- | --- | --- | --- | --- | --- | --- | --- | --- | --- | --- | --- | --- | --- | --- | --- | --- | --- | --- | --- | --- | --- | --- | --- | --- | --- | --- | --- | --- | --- | --- | --- | --- | --- | --- | --- | --- | --- | --- | --- | --- | --- | --- | --- | --- | --- | --- | --- | --- | --- | --- | --- | --- | --- | --- | --- | --- | --- | --- | --- | --- | --- | --- | --- | --- | --- | --- | --- | --- | --- | --- | --- | --- | --- | --- | --- | --- | --- | --- | --- | --- | --- | --- | --- | --- | --- | --- | --- | --- | --- | --- | --- | --- | --- | --- | --- | --- | --- | --- | --- | --- | --- | --- | --- | --- | --- | --- | --- | --- | --- | --- | --- | --- | --- | --- | --- | --- | --- | --- | --- | --- | --- | --- | --- | --- | --- | --- | --- | --- | --- | --- | --- | --- | --- | --- | --- | --- | --- | --- | --- | --- | --- | --- | --- | --- | --- | --- | --- | --- | --- | --- | --- | --- | --- | --- | --- | --- | --- | --- | --- | --- | --- | --- | --- | --- | --- | --- | --- | --- | --- | --- | --- | --- | --- | --- | --- | --- | --- | --- | --- | --- | --- | --- | --- | --- | --- | --- | --- | --- | --- | --- | --- | --- | --- | --- | --- | --- | --- | --- | --- | --- | --- | --- | --- | --- | --- | --- | --- | --- | --- | --- | --- |
|  |  | |
|  |  | |
|  |  | |
|  |  | |
|  |  | |
|  |  | |
|  |  | |
|  |  | |
|  |  | |
