# Supplementary material for: Flavonoid Library Screening Reveals Kaempferol as a Potential Antiviral Agent Against African Swine Fever Virus
Source: Front Microbiol. 2021 Oct 21;12:736780. doi: 10.3389/fmicb.2021.736780 (PMC8567988; doi:10.3389/fmicb.2021.736780)
Supplement: Supplementary file 2 [file Table_2.pdf]

Column1	Column2	Column3	Column5	Column6	Column8	Column9	Column12
	EXP1		EXP2		EXP3		AVE
	**RFU**						
	**12h**	**24h**	**12h**	**24h**	**12h**	**24h**	
**Control**	2177	2045	2131	2245	2234	1970	
**Rap**	3657	3619	3802	3883	3530	3585	
**Kae**	3159	3027	3068	3255	2926	2975	
**ASFV**	1962	2372	1854	2357	1809	2502	
**ASFV+Kae**	3001	2340	2876	2626	2837	2561	
	**Fold change**						
	**12h**	**24h**	**12h**	**24h**	**12h**	**24h**	**12h**
**Control**	1	1	1	1	1	1	1
**Rap**	1.68	1.77	1.78	1.73	1.58	1.82	1.68
**Kae**	1.45	1.48	1.44	1.45	1.31	1.51	1.4
**ASFV**	0.9	1.16	0.87	1.05	0.81	1.27	0.86
**ASFV+Kae**	1.37	1.14	1.35	1.17	1.27	1.3	1.33

Column13
**24h**
1
1.77333333
1.48
1.16
1.20333333
